# Influence of Coping, Esteem, and Resilience on Caregiver Distress in Pancreatic Cancer Patient–Caregiver Dyads

**DOI:** 10.3390/healthcare13020114

**Published:** 2025-01-09

**Authors:** Nicole Nardella, Brent Taiting Xia, Kelvin Allenson, Adrianna Oraiqat, Wenyi Fan, Qianxing Mo, Jennifer Permuth, Dae Won Kim, Pamela Hodul

**Affiliations:** 1 Department of Gastrointestinal Oncology, Moffitt Cancer Center, Tampa, FL 33612, USA; brent.xia@stelizabeth.com (B.T.X.); kcallenson@houstonmethodist.org (K.A.); adrianna.oraiqat@moffitt.org (A.O.); jenny.permuth@moffitt.org (J.P.); daewon.kim@moffitt.org (D.W.K.); pamela.hodul@moffitt.org (P.H.); 2 Division of Surgical Oncology, St. Elizabeth Healthcare, Edgewood, KY 41017, USA; 3 Division of Surgical Oncology, Houston Methodist Hospital, Houston, TX 77030, USA; 4 Biostatistics and Bioinformatics Shared Resource, Moffitt Cancer Center, Tampa, FL 33612, USA; wenyi.fan@moffitt.org (W.F.); qianxing.mo@moffitt.org (Q.M.); 5 Department of Cancer Epidemiology, Moffitt Cancer Center, Tampa, FL 33612, USA

**Keywords:** caregiver coping, approach coping, avoidant coping, resilience, self-esteem, caregiver burden, cancer caregiving, pancreatic cancer

## Abstract

**Background/Objectives:** Through survey and analysis of pancreas cancer patient–caregiver dyads, we aimed to identify patient and caregiver characteristics that influence and determine the impact of caregiver coping strategies, self-esteem, and resilience on caregiver distress. **Methods:** This was a cross-sectional, observational study including pancreatic cancer patients and their caregivers. Demographics of patients and caregivers were collected. Caregivers completed validated instruments (National Comprehensive Cancer Network (NCCN) Distress Thermometer, Caregiver Reaction Assessment (CRA), Perceived Stress Scale 4 (PSS-4), Patient Reported Outcomes Measurement Information System-Anxiety/Depression Short Form (PROMIS-Anxiety/Depression), Brief Resilience Scale (BRS), Zarit Caregiver Burden Interview (CBI-12), and Brief Coping Orientation to Problems Experienced (COPE)) investigating anxiety, depression, perceived stress, caregiver burden, use of approach and avoidant coping, resilience, and self-esteem. Descriptive statistics, univariate, and multiple linear regression models were used to analyze the data. **Results:** One hundred and fourteen patient–caregiver dyads were included in this study. The majority of patients were male (55%), 65% of caregivers were female, and 64% of patients were receiving palliative intent treatment. Younger caregiver age, more personal care tasks, higher patient distress, and caregiving for a parent were characteristics related to caregiver avoidant coping. Fewer caregiving activities and early clinical stage were associated with caregiver approach coping. High caregivers’ self-esteem was significantly associated with fewer personal care tasks to perform and with caregivers of patients with higher levels of education. Avoidant coping and low resilience were both significantly correlated to distress, anxiety, depression, caregiver burden, and perceived stress. Additionally, low self-esteem was associated with a high perceived caregiver burden. **Conclusions:** Overall, caregiver factors such as age, relationship with the patient, and number of care tasks and activities influence caregivers coping and self-esteem. Additionally, patient education and clinical stage impacted caregiver coping and self-esteem. Developing interventions to address caregiver coping, self-esteem, and resilience will prove beneficial in improving caregiver distress, anxiety, depression, burden, and perceived stress.

## 1. Introduction

Caregivers are defined as any relative, partner, friend, or neighbor who has a significant personal relationship with and provides a broad range of assistance for a person with a chronic or disabling condition [1]. Assistance may come in the form of time and logistics, physical care, emotional burden, and financial support [2]. Often, caregiving limits the caregiver’s ability to participate in regular social activities and work [3]. With the addition of these caregiving activities, caregivers frequently delay their own healthcare needs, which can negatively influence their well-being [4]. This heightened level of stress and burden associated with caregiving can result in anger, depression, and anxiety [5].

According to the American Cancer Society, approximately 40.9% of men and 39.1% of women will be diagnosed with cancer of any site at some point in their lifetime [6]. With more than one in three persons afflicted by a cancer diagnosis, the number of individuals expected to care for them is equivalent. Caring for someone with cancer adds an additional layer of burden, both physically and emotionally. Cancer caregiving tasks include monitoring for treatment-related side effects, assisting with treatment decision-making, administering medication, helping to manage symptoms (pain, nausea, and fatigue), providing transportation to doctors’ appointments, performing personal care tasks (feeding, grooming, etc.), and helping with daily life activities (managing finances, preparing meals, etc.) [7].

Caregiving in adenocarcinoma pancreatic cancer (PC) is particularly challenging. Patients with PC have a high symptom burden, and progression is often rapid, with less than 12% of patients living 5 years [6]. More than half of patients present with advanced stage, and survival for those with metastatic disease is 6–12 months [8]. Over 80% of patients experience pain and gastrointestinal symptoms, resulting in caregivers attempting to manage the significant emotional stressors related to this high symptom burden [9]. Despite numerous studies on caregivers in general and within the context of cancer, few investigators have studied and published on the caregiving experience of patients with PC. Preparation and adjustment for both patients and their caregivers are limited and present specific challenges that need to be identified [2]. Reported studies have focused mainly on quality of life (QOL) and emotional stressors encountered by the caregiver, while a handful of studies have addressed depression and emotional stress in PC patients [2,10,11,12,13,14,15]. A significant gap in the literature exists in the identification of opportunities for intervention and emotional support for PC caregivers.

Our group has previously identified PC caregiver characteristics that were significant predictors of caregiver anxiety, depression, distress, and perceived burden—mainly young caregiver age, number of caregiving activities, employment status, and caregiver poor health status [16]. These stressors impact caregiver QOL and influence caregiver coping mechanisms, self-esteem, and resilience. Coping strategies include approach coping (actively moving towards a stressor in order to resolve an issue) and avoidant coping (actively moving away from a stressor in order to resolve an issue). Caregiver resilience and positive coping strategies have been shown to be protective and constructive to caregiver QOL and management of stress [17]. Caregiver coping mechanisms, self-esteem, and resilience have a direct relationship with patient care, though there is a paucity of studies investigating the impact on caregiver overall well-being and mental health specifically in relation to PC [17,18,19,20,21]. We aimed to identify the role of coping, self-esteem, and resilience on caregiver distress, anxiety, depression, and burden to address this knowledge deficit. Identification of caregiver factors that positively impact coping, self-esteem, and resilience will assist in the development of future interventions to improve PC caregiver well-being.

## 2. Materials and Methods

### 2.1. Participants and Procedure

This study was conducted at Moffitt Cancer Center (2020–2021). Questionnaire data for this analysis were gathered using an IRB-approved (Advarra Pro00040381) cross-sectional, observational study of the psychosocial well-being of PC patients and their identified caregivers. All participating patients were approached and consented to Total Cancer Care, our institutional biorepository. Patient electronic health records were used to collect demographic and clinical data.

Patient eligibility included adult with any stage of PC diagnosis. Patients were recruited any time after diagnosis, given they had multiple consultations at our institution. Eligibility criteria for caregivers included an adult individual who self-identifies as a current caregiver of an eligible patient [16]. All types of caregivers were considered; however, our cohort consisted of all informal primary/secondary caregivers. Eligible patient–caregiver dyads were approached by convenience sampling during clinic visits or by mailed letter if they were not able to be approached. A follow-up telephone call was conducted within two weeks of their initial clinic consultation to confirm receipt of the letter. Electronic or paper questionnaires were then completed based on modality preference. Electronic versions were completed at home through emailed links, while paper versions were completed either in clinic or at home (sent via mail with a prepaid return envelope). Patients and caregivers completed independent questionnaires at the same time point. Upon completion of the surveys, the study coordinator assigned participants study IDs to deidentify the data for analysis. The current analysis focuses on caregiver data only, as our previous publication was in relation to the patient data.

### 2.2. Measures

#### 2.2.1. Demographics and Caregiver Activities

Caregivers self-reported demographic information, including socioeconomic status, age, language, gender, education, relationship to patient, health status, and employment status. Survey analysis included the number of caregiving activities in relation to personal care (i.e., feeding, grooming, etc.), daily life activities (i.e., managing finances, preparing meals, etc.), and other activities (i.e., emotional support, appointment scheduling, etc.).

#### 2.2.2. Avoidant and Approach Coping Styles

The Brief Coping Orientation to Problems Experienced (COPE), a 28-item measure, was used to report caregiver coping mechanisms—specifically avoidant and approach coping styles [22]. This instrument uses a Likert scale with a response range of 1 (“I haven’t been doing this at all”) to 4 (“I’ve been doing this a lot”). This validated instrument measured any behavioral, cognitive, and emotional activity in relation to avoidant and approach coping. Sample statements included “I’ve been giving up trying to deal with it” and “I’ve been taking action to try to make the situation better”.

#### 2.2.3. Self-Esteem

The Caregiver Reaction Assessment (CRA) was administered to caregivers to assess self-esteem. A subscale of seven items (questions 1, 7, 9, 12, 17, 20, and 23) specific to self-esteem was measured and was reported as a continuous variable. Instrument responses were on a Likert scale with a range of 1 (strongly disagree) to 5 (strongly agree). Higher overall scores equated to contentment and self-esteem in the caregiving process [23].

#### 2.2.4. Resilience

The Brief Resilience Scale (BRS), a 6-item measure, was used to assess resilience [24]. Instrument responses were on a Likert scale ranging from strongly agree to strongly disagree. Overall scores ranged from 6 to 30. Sample questions included “I tend to bounce back quickly after hard times” and “I have a hard time making it through stressful events”.

#### 2.2.5. Distress

Patient and caregiver distress were gauged using the National Comprehensive Cancer Network (NCCN) Distress Thermometer. This validated instrument measured subjective distress experienced by the patient and caregiver in the previous week. Distress was measured on a scale ranging from 0 (no distress) to 10 (extreme distress). For scores ≥ 4, a problem list of key issues of concern (practical, family, emotional, spiritual/religious, and physical concerns) was reviewed for further assessment [25].

#### 2.2.6. Anxiety and Depression

The 4-item Patient Reported Outcomes Measurement Information System-Anxiety Short Form 4a (PROMIS-Anxiety) was used to determine caregiver anxiety, while the Patient Reported Outcomes Measurement Information System–Depression Short Form 4a (PROMIS-Depression) was used to determine depression. These tools assessed a 7-day time frame with a 5-point Likert scale frequency (1 = never, 2 = almost never, 3 = sometimes, 4 = fairly often, and 5 = very often) to score anxiety and depression [26]. Higher scores correlated with higher anxiety and depression. Sample questions included “My worries overwhelm me” for anxiety and “I felt worthless” for depression.

#### 2.2.7. Caregiver Burden

The 12-item Zarit Caregiver Burden Interview (CBI-12) was used to study caregiver burden. This instrument utilized a Likert scale with the prompt “Do you feel…” and responses ranging from 0 (never) to 4 (nearly always). Overall scores ranged from 0 to 48, with scores ≥ 17 indicating high burden [27]. Sample question included, “Overall, how burdened do you feel in caring for (care recipient)?”.

#### 2.2.8. Perceived Stress

The 4-item Perceived Stress Scale 4 (PSS-4) was used to measure global levels of perceived stress. Responses rated how often the caregiver experienced stressful situations in the previous month [28]. Responses ranged from 0 (never) to 4 (very often), with overall scores ranging from 0 to 16. Higher scores indicated more caregiver-perceived stress [29].

### 2.3. Data Analysis

Descriptive statistics were used to report patient and caregiver sociodemographic information. Continuous variables were summarized by median with range, and categorical variables were reported with frequency and percentage. Patient demographics and clinical stage, as well as caregiver demographics and caregiving activities, were analyzed as predictors of caregiver coping, esteem, and resilience using univariate and multiple linear regression models. Caregiver coping and esteem scores and the resilience scale were analyzed to predict caregiver distress using univariate and multiple linear regression models. The predictors with a *p*-value < 0.05 in the univariate linear models were kept in the multiple linear models. *p*-values were two-sided, and *p*-values < 0.05 were considered statistically significant. All statistical analyses were performed using R (version 4.1.2).

## 3. Results

### 3.1. Demographics

A total of 114 patient–caregiver dyads were enrolled. Demographics and clinical characteristics are displayed in Table 1. The median age of patients and caregivers enrolled was 69 and 66, respectively. The patient sub-cohort comprised 45% females and 55% males, while the caregiver sub-cohort comprised 65% females and 35% males. Notable was that 72% (n = 13) of caregivers who were not spouses/partners (n = 18) were female. The majority of patients (44%) and caregivers (57%) were high-school graduates, went to vocational school, or had some college education. Resectable (n = 10) and borderline resectable (n = 21) patients received curative treatment intent (36%), while locally advanced (n = 29) and metastatic (n = 44) patients received palliative treatment intent (64%). Approximately 84% of caregivers were the patient’s spouse/partner. Most caregivers (59%) reported themselves as having excellent/very good health. The majority of caregivers either lived with the patient, were the primary/only caregiver, and/or did not have paid help. Most caregivers were not employed (59%). The median number of caregiving hours/week was 20.

### 3.2. Avoidant Coping

Avoidant coping style was analyzed from a subset of the Brief COPE. Univariate analysis showed younger caregiver age (*p* = 0.022), more personal care tasks (*p* = 0.038), higher patient NCCN distress thermometer scores (*p* = 0.016), the caregiver being the patient’s child (*p* = 0.042), and indicating caregiving affected employment status (*p* = 0.005) correlated with higher avoidant coping scores. On multiple linear regression analysis, only caregiving affecting employment status correlated with higher avoidant coping scores with a *p*-value of 0.042 when controlling for the caregiver’s age, total tasks of caregiving, and relationship to patients (Table 2).

### 3.3. Approach Coping

Approach coping style was analyzed from a subset of the Brief COPE. Univariate analysis showed more other caregiving activities correlated with higher approach coping scores (*p* = 0.047). Non-metastatic PC clinical stage significantly correlated with higher approach coping scores (*p* = 0.009). Additionally, caregivers with better overall health correlated with high approach coping scores on univariate analysis (*p* = 0.017). Multiple linear regression models, including caregiving and other activities, patient clinical stage, and caregiver overall health, showed patient clinical stage and caregiver overall health had a significant relationship with approach coping scores, with *p*-values of 0.015 and 0.026 (Table 2).

### 3.4. Self-Esteem

The CRA esteem subscale showed that fewer caregiver personal care total tasks were associated with higher caregiver esteem scores on univariate analysis (*p* = 0.003). Univariate and multiple linear regression analyses showed higher patient education (college graduate) were significantly related to higher caregiver self-esteem with *p*-values of 0.012 and 0.017, respectively (Table 2).

### 3.5. Resilience

Analysis showed no significant relationships between independent variables and the BRS.

### 3.6. Distress

Caregivers with higher avoidant coping significantly correlated with higher caregiver NCCN distress thermometer scores (*p* < 0.001). Lower resilience scores were also significantly correlated with higher caregiver NCCN distress thermometer scores on univariate analysis (*p* < 0.001). These findings remained significant in multiple linear regression models, with *p*-values of <0.001 and 0.007, respectively (Table 3).

### 3.7. Anxiety and Depression

Caregivers with higher avoidant coping use were significantly correlated with higher caregiver PROMIS-Anxiety scores, with a *p*-value of <0.001. Lower resilience scores were also significantly related to higher caregiver PROMIS-Anxiety scores (*p* < 0.001). This maintained significance with multiple linear regression models, with *p*-values of <0.001 and 0.003, respectively.

Caregivers with higher avoidant coping use were significantly correlated with higher caregiver PROMIS-Depression scores, with a *p*-value of <0.001. Lower resilience scores were also significantly related to higher caregiver PROMIS-Depression scores (*p* < 0.001). This maintained significance with multiple linear regression models, with both *p*-values < 0.001 (Table 3).

### 3.8. Caregiver Burden

Caregivers with lower self-esteem scores (*p* = 0.001) and higher avoidant coping scores (*p* < 0.001) were significantly correlated with higher caregiver Zarit scores. Lower resilience scores were also significantly related to higher caregiver Zarit scores on univariate analysis (*p* = 0.002). When putting self-esteem, avoidant coping, and resilience scores together in multiple linear regression models, low self-esteem and avoidant coping scores remained significant in the model with *p*-values of 0.004 and <0.001, respectively (Table 3).

### 3.9. Perceived Stress

Caregivers with higher avoidant coping use were significantly correlated with higher caregiver PSS-4 scores (*p* < 0.001). Lower resilience scores were also significantly related to higher caregiver PSS-4 scores on univariate analysis (*p* < 0.001). Avoidant coping remained significant with a *p*-value < 0.001 when controlling for resilience scores (Table 3).

## 4. Discussion

In this single-institution, cross-sectional, observational study of PC patient–caregiver dyads, several key demographic and clinical factors were strongly associated with caregiver response to caring for someone with PC. Our results demonstrated that the majority of caregivers were white, non-Hispanic females, particularly for patients who did not have a spouse/partner caregiver. Previous work has shown that women comprise approximately 70% of caregivers, regardless of whether they are full-time employed or not [30]. Caregivers self-reported to be in excellent/very good health. The median age of caregivers was 66 years, most reporting that they were not employed. Though we did not put a restraint on what type of caregivers were eligible for this study, the overwhelming majority were spouses/partners of the patient and did not have additional paid help to assist in caregiving. The cohort consisted of mostly patients receiving palliative intent treatment. Previous work showed advanced stage often corresponded with physiological and psychosocial burden and diminished spiritual wellness [31]. This discussion explores significant caregiver characteristics as they relate to caregiver coping, self-esteem, and resilience.

Caregiver use of avoidant coping predicted increased anxiety, depression, distress, perceived stress, and burden. Tan et al. illustrate that utilizing avoidant coping strengthens and prolongs negative emotional states of stress and anxiety, and others have suggested that this leads to little or no effectiveness with handling situations [32,33]. The lack of caregivers addressing their own needs by distracting and avoiding situations is associated with perceived burden, increased depression, and less satisfaction [34]. Our findings indicated that higher avoidant coping was found in younger caregivers and when children are caregivers of the patient. Previous work by Mishra et al. in reference to young adult (age 23–39) caregiving showed that caregivers looked to escapism and poor handling of coping, further supporting our findings [35]. Higher avoidant coping was found when caregivers provided a higher number of personal/cancer care tasks, cared for a patient with high distress, and were employed during the period of caregiving. For the latter, fewer working hours, decreased income, and increased household obligations can result in additional stressors and negatively impact their psychosocial well-being [36].

Approach coping was not a significant factor for caregiver distress, anxiety, depression, perceived stress, or burden and is a preferred coping mechanism to avoidant coping. Significant characteristics of caregivers utilizing approach coping were fewer activities to perform and better health status. Caregivers with better health status may be more equipped to handle situations rather than use avoidance. Caregivers must be cautious, however, not to neglect their own healthcare, as this has been linked to negative effects on physical and psychological well-being [36]. Additionally, approach coping was more likely to be associated with caregivers of patients undergoing treatment with curative intent. A study comparing caregivers of patients with curative intent vs. palliative intent treatment demonstrated that the palliative intent caregivers had lower QOL scores and worse overall health [37], suggesting that the stage of disease can influence the caregiver’s experience and health, as well as their ability to cope.

According to Yang et al., higher levels of self-esteem are associated with effective coping strategies, such as approach coping. Furthermore, effective coping strategies have been linked to decreased caregiver burden [38]. Our study similarly demonstrated that caregivers with high self-esteem, particularly those with less personal tasks, were more likely to report less burden. While previous studies have reported that caregiver education level was linked to self-esteem, our study did not find any significance [39]. Our results, however, did suggest that patient education level may be a contributing factor to caregiver self-esteem, with higher patient education correlating to higher caregiver self-esteem. This may be due to the patient’s having higher health literacy and greater ability to understand diagnosis/treatment details, as well as prescriptions and appointments. In a study of stroke patients, it was found that patient care education reduced the burden of care and improved QOL for caregivers [40]. Hence, findings demonstrate that patient knowledge is critical to improving caregiver self-esteem and burden.

When considering resilience, our results found that caregivers with low resilience were more likely to experience distress, anxiety, depression, perceived stress, and burden. Palacio et al. demonstrated that resiliency resulted in less emotional distress and less caregiver burden in patients with chronic, advanced disease and end-of-life stages [17]. Furthermore, they showed that resiliency had a positive impact on QOL for caregivers. These findings suggest that when faced with adversity, caregivers with higher resilience were more likely to experience fewer psychosocial effects.

Inherent to the nature of a cross-sectional, observational study, there are several limitations. Patient–caregiver dyads consented, and survey collection was at a singular time point, which may not fully capture variations in distress, psychosocial burden, and adaptive mechanisms that may occur throughout the disease and treatment process. Due to this inability to establish causality through lack of tracking over time, future work focused on longitudinal studies may address this limitation. Additionally, it is well recognized that coping strategies and resiliency are heavily influenced by cultural, religious, and societal factors. Our sample was relatively homogeneous regarding race/ethnicity, a reflection of our tertiary care and referral patterns, which may have sampling bias. Our findings may not be scalable to more culturally diverse patient–caregiver dyads, as well as those who receive care internationally, where there may be differences in the economics of healthcare and delivery compared to a tertiary referral center in the United States. Lastly, there may be a selection bias and unmeasured difference in psychosocial characteristics and level of burden/distress between patients and caregivers that agreed to participate in this study and those that did or did not complete the survey.

## 5. Conclusions

In conclusion, an improved understanding of caregiver factors related to psychosocial well-being provides insight into combating caregiver burden in patients with PC. Caregiver age, employment status, and overall health are significant factors in caregiver coping, self-esteem, and resilience. In addition, patient education may play a key role in sharing responsibilities related to disease management.

The implementation of resources to enhance caregiver wellness may improve outcomes for both the caregiver and patient. Promoting environments for caregivers to utilize healthy coping strategies, such as approach coping, may alleviate distress, anxiety, depression, perceived stress, and burden. Interventions to endorse an approach to coping may lead to higher levels of self-esteem and resilience. Examples include the implementation of assistance programs such as in-home aids, errand runners, and/or respite care to reduce the number of caregiving tasks. Mobile applications focused on mental well-being (mindfulness, meditation, and journaling) and physical health offer convenient outlets to promote cognitive coping skills and emotional coping mechanisms. Additionally, implementation of health literacy programs, support groups, and coaching for patients and caregivers may further decrease psychosocial stressors and enhance social coping skills. Future studies will be needed to assess the impact of such interventions on caregivers of patients with PC.

## Figures and Tables

**Table 1:** Demographics and clinical characteristics.

	Patient	Caregiver
	n = 114	n = 114
Age	69 (35–87)	66 (33–88)
Race		
Asian	3 (2.63%)	3 (2.63%)
Black	4 (3.51%)	3 (2.63%)
Native Hawaiian/Pacific Islander	1 (0.88%)	0 (0.00%)
White/Caucasian	106 (92.98%)	108 (94.74%)
Hispanic		
Yes	7 (6.14%)	7 (6.14%)
No	104 (91.23%)	107 (93.86%)
Unknown	3 (2.63%)	0 (0.00%)
Gender		
Female	51 (44.74%)	74 (64.91%)
Male	63 (55.26%)	40 (35.09%)
Education		
College graduate (bachelor’s degree or higher)	43 (37.72%)	46 (40.35%)
High-school graduate/vocational school/some college	51 (44.74%)	65 (57.02%)
Unknown	20 (17.54%)	3 (2.63%)
Patient Clinical Stage		
Resectable	10 (8.77%)	
Borderline Resectable	31 (27.19%)	
Locally Advanced	29 (25.44%)	
Metastatic	44 (38.60%)	
Treatment Status		
Curative	41 (35.96%)	
Palliative	73 (64.04%)	
Time Since Diagnosis (months)	6 (0–86)	
Age-Adjusted Charlson Comorbidity Score	5 (0–15)	
ECOG Score		
0–1	106 (92.98%)	
2–3	5 (4.39%)	
Unknown	3 (2.63%)	
Caregiver Overall Health		
Excellent/Very Good		67 (58.77%)
Good/Fair		47 (41.23%)
Relationship to Patient		
Spouse/partner		96 (84.21%)
Child		10 (8.77%)
Other		8 (7.02%)
Lives with patient?		
Yes		101 (88.60%)
No		13 (11.40%)
Years of relationship with patient		38 (2–60)
Months of caregiving		8 (0–684)
Caregiving Arrangement		
Primary and only caregiver		82 (71.93%)
Primary caregiver, with some help from others		23 (20.18%)
Secondary caregiver		3 (2.63%)
Split caregiving equally with others (multiple caregivers)		4 (3.51%)
Caregiver Paid Help In Addition to Caregiver		
Yes		5 (4.39%)
No		109 (95.61%)
Caregiver Household Income		
Less than USD 50,000		28 (24.56%)
USD 50,000–USD 99,999		19 (16.67%)
USD 100,000 or more		29 (25.44%)
Unknown		38 (33.33%)
Caregiver Employment Status		
Employed		44 (38.60%)
Not employed		67 (58.77%)
Unknown		3 (2.62%)
Caregiving Hours/Week		20 (0–168)
Caregiving Personal Care, # tasks		0 (0–8)
Caregiving Daily Activities, # tasks		5 (0–7)
Caregiving Other Activities, # tasks		7 (1–9)

Values reported as median (range) where applicable. Number of tasks is indicated as # tasks.

**Table 2:** Univariate and multiple linear regression models.

Variable	Univariate Model	*p*-Value	Multiple Linear Regression Model	*p*-Value	Overall *p*-Value
	Mean/β (95% CI)		Mean/β (95% CI)		
Avoidant Coping					
Caregiver age	−0.012 (−0.023, −0.002)	0.022	0.00 (−0.014, 0.013)	0.947	
Caregiver personal care total tasks	0.060 (0.003, 0.116)	0.038	0.036 (−0.020, 0.092)	0.204	
Patient distress thermometer score	0.057 (0.011, 0.102)	0.016	0.041 (−0.004, 0.086)	0.076	
Caregiver relationship to patient		0.042			
Child	3.42 (2.94, 3.89)		1.0 (Reference)	-	0.173
Spouse/partner	2.88 (2.75, 3.01)		−0.419 (−0.886, 0.049)	0.079	
Other	2.81 (2.32, 3.30)		−0.504 (−1.106, 0.098)	0.100	
Caregiving affected employment status		0.005			
No	2.80 (2.66, 2.94)		1.0 (Reference)	-	
Yes	3.17 (2.95, 3.40)		0.301 (0.012, 0.590)	0.042	
Approach Coping					
Caregiver other activities total tasks	0.110 (0.001, 0.218)	0.0474	0.086 (−0.018, 0.191)	0.105	
Patient clinical stage		0.009			
Localized (resectable, borderlineresectable, or locally advanced)	5.43 (5.16, 5.70)		1.0 (Reference)	-	
Metastatic	4.84 (4.48, 5.20)		−0.535 (−0.963, −0.106)	0.015	
Caregiver overall health		0.017			
Excellent/very good	5.42 (5.13, 5.70)		1.0 (Reference)	-	
Good/fair/poor	4.88 (4.55, 5.21)		−0.485 (−0.910, −0.060)	0.026	
Self-Esteem					
Caregiver personal care total tasks	−0.073 (−0.120, −0.026)	0.003	−0.036 (−0.082, 0.010)	0.123	
Patient education		0.012			
College graduate(bachelor’s or higher)	4.31 (4.15, 4.47)		1.0 (Reference)	-	
High-schoolgraduate/vocationalschool/some college	4.56 (4.44, 4.69)		0.239 (0.044, 0.434)	0.017	

Note: β is the slope of the linear model for continuous variables and the mean for categorical variables.

**Table 3:** Univariate and multiple linear regression models.

Variable	Univariate Model Mean/β (95% CI)	*p*-Value	Multiple Linear Regression Model Mean/β (95% CI)	*p*-Value	Overall *p*-Value
Caregiver NCCN Distress Thermometer					
Avoidant Coping	1.815 (1.153, 2.478)	<0.001	1.392 (0.688, 2.097)	<0.001	
BRS Total Score		<0.001			
Low resilience	7.50 (6.55, 8.45)		1.0 (Reference)	-	0.007
Normal resilience	4.38 (3.85, 4.91)		−2.204 (−3.571, −0.836)	0.002	
High resilience	3.92 (2.72, 5.12)		−2.237 (−3.859, −0.614)	0.007	
PROMIS-Anxiety					
Avoidant Coping	7.270 (4.919, 9.622)	<0.001	5.554 (3.073, 8.034)	<0.001	
BRS Total Score		<0.001			
Low resilience	66.8 (61.3, 72.4)		1.0 (Reference)	-	0.003
Normal resilience	55.0 (53.3, 56.7)		−8.160 (−12.975, −3.344)	0.001	
High resilience	52.3 (47.8, 56.8)		−9.177 (−14.890, −3.464)	0.002	
PROMIS-Depression					
Avoidant Coping	7.224 (5.215, 9.232)	<0.001	5.403 (3.327, 7.478)	<0.001	
BRS Total Score		<0.001			
Low resilience	61.6 (56.4, 66.8)		1.0 (Reference)	-	<0.001
Normal resilience	50.9 (49.3, 52.6)		−7.128 (−11.158, −3.098)	0.001	
High resilience	46.5 (43.6, 49.3)		−9.932 (−14.713, −5.151)	<0.001	
Zarit Score					
CRA Self-Esteem Score	−4.002 (−6.342, −1.662)	0.001	−3.087 (−5.186, −0.988)	0.004	
Avoidant Coping	5.338 (3.521, 7.155)	<0.001	4.335 (2.390, 6.280)	<0.001	
BRS Total Score		0.002			
Low resilience	15.40 (11.3,19.4)		1.0 (Reference)	-	0.309
Normal resilience	10.10 (8.47,11.6)		−2.158 (−5.909, 1.594)	0.257	
High resilience	7.12 (4.24,10)		−3.455 (−7.902, 0.991)	0.126	
PSS-4 Score					
Avoidant Coping	2.569 (1.911, 3.226)	<0.001	2.296 (1.575, 3.016)	<0.001	
BRS Total Score		<0.001			
Low resilience	7.36 (6.06, 8.65)		1.0 (Reference)	-	0.205
Normal resilience	4.88 (4.30, 5.46)		−0.965 (−2.365, 0.434)	0.174	
High resilience	3.64 (2.31, 4.97)		−1.502 (−3.163, 0.158)	0.076	

Note: β is the slope of the linear model for continuous variables and the mean for categorical variables.

## Data Availability

The raw data supporting the conclusions of this article will be made available by the authors upon reasonable request.
